# Obesogens in Adolescence: Challenging Aspects and Prevention Strategies

**DOI:** 10.3390/children11050602

**Published:** 2024-05-16

**Authors:** Marina Nicolaou, Meropi Toumba, Alexandros Kythreotis, Habib Daher, Nicos Skordis

**Affiliations:** 1 Barts and The London School of Medicine and Dentistry, Queen Mary University of London, London E1 4NS, UK; m.nicolaou@smd19.qmul.ac.uk (M.N.); a.k.kythreotis@smd19.qmul.ac.uk (A.K.); 2 Paediatric Endocrinology Clinic, Department of Paediatrics, Aretaeio Hospital, 2024 Nicosia, Cyprus; meropi.toumba@doctors.org.uk; 3 School of Medicine, University of Nicosia, 2414 Nicosia, Cyprus; daher.hab@live.unic.ac.cy; 4 Division of Paediatric Endocrinology, Paedi Center for Specialized Paediatrics, 2025 Nicosia, Cyprus

**Keywords:** endocrine disrupting chemicals, obesogens, obesity, children, adolescents, prevention strategies

## Abstract

Childhood obesity has become a global epidemic, with significant increases in prevalence over recent decades. While excessive calorie consumption and physical inactivity are known factors, emerging research highlights the role of endocrine-disrupting chemicals (EDCs), particularly obesogens, in obesity’s pathogenesis. This review explores the historical context of the environmental obesogens hypothesis, their sources, mechanism of action, impact on prenatal and postnatal development, and epigenetics. Additionally, it discusses the long-term consequences of childhood obesity and proposes prevention strategies that will mitigate negative health effects. Obesogens were found to disrupt hormonal balance and metabolic processes through various mechanisms such as altering gene expression, hormonal interference, and inflammation. Especially significant was exposure during critical windows of development, which correlates with an increased risk of obesity in childhood or adolescence. Long-term effects of childhood obesity include chronic health conditions and psychosocial issues. A comprehensive approach is necessary to address childhood obesity encompassing genetic, environmental, and lifestyle factors. Prevention strategies should focus on reducing obesogen exposure, promoting healthy lifestyles, and implementing regulatory policies. Future research should investigate obesogens–diet interactions, microbiome impacts, and combined obesogens effects. Long-term human studies are also crucial for validating findings from animal models and allowing for informed decision-making to combat the obesity pandemic.

## 1. Introduction

Childhood obesity is defined by the Centers for Disease Control and Prevention (CDC) as a body mass index (BMI) at or above the 95th percentile, or +2 SDs for age and sex. For children younger than 5 years, it is defined as weight for height above +3 SDs. Overweight is defined by a BMI above the 85th percentile or +1 SDs, but less than the 95th percentile; similarly, for children younger than 5 years, it is defined as weight for height above +2 SDs. Obesity has increased dramatically over the last fifty years, giving rise to a pervasive and harmful public health crisis, frequently denoted as an epidemic [1,2]. According to NCD Risk Factor Collaboration (NCD-RisC), the worldwide age-standardized prevalence of obesity in children and adolescents aged 5–19 years rose from 0.9% to 7.8% for boys and from 0.7% to 5.6% for girls between 1975 and 2016 [3]. Furthermore, in 2019, the World Obesity Federation estimated that by 2025, around 390 million children and adolescents would be overweight and 206 million would be obese, with an expected increase to 254 million by 2030 [4]. As childhood obesity becomes more prevalent worldwide, so does the burden of comorbidities associated with it. It is not just a matter of body image and cosmetic concerns, but it is strongly correlated with increased risk for type 2 diabetes, heart diseases, and other metabolic diseases [5]. While numerous theories have tried to explain this rise in obesity worldwide, there remains considerable uncertainty. Although non-lifestyle factors such as genetic predispositions are known to be associated with obesity, a recent analysis of 97 BMI-associated loci found that these only account for ~2.7% of BMI variation [6]. Thus, it can be reasonably concluded that abrupt changes in the gene pool, of a magnitude required to be the primary cause of the current issue, are unlikely factors. The prevailing medical explanation continues to be that obesity is due to a combination of excessive caloric consumption and insufficient energy expenditure [7]. Despite a well-recognised global rise in the consumption of fat and sugar-rich food, as well as a surge in physical inactivity secondary to sedentary lifestyles, this dramatic increase in obesity rates over a relatively short period cannot be explained by energy imbalances alone [7]. Consequently, additional mechanisms have been proposed to contribute to the modern obesity epidemic, one of which is exposure to endocrine-disrupting chemicals (EDCs). The Endocrine Society defines EDCs as external chemicals that can mimic, block, or interfere with the way the body’s hormones work [8]. A subset of these chemicals, known as “obesogens” have been found to play an important role in the pathogenesis of obesity, especially if exposure occurs during critical windows of development [9]. Identifying the sources of obesogens and understanding their pathophysiology will allow us to implement more effective prevention strategies and better treatment approaches.

## 2. History of Obesogens and the Environmental Obesogens Hypothesis

The relationship between EDCs and obesity first gained attention in the early twenty-first century and more specifically in 2002, when Dr. Paula Baillie-Hamilton authored the first review paper on environmental chemicals and their effect on obesity [10]. She proposed a correlation between the actual obesity epidemic and the increased production of exogenous chemicals following World War II. This link was particularly evidenced by a figure in her review illustrating an increase in obesity with the increase in the production of chemicals throughout the course of several decades [10]. In 2006, Felix Grun and Bruce Blumberg introduced the idea of environmental disruptors and published a paper revealing that tributyltin (TBT), a xenobiotic compound utilized in marine paints, wood preservation, and industrial water systems, induced weight gain in a population of mice [11,12]. This reinforced the concept that these chemicals could potentially lead to obesity by interfering with normal development and homeostatic control of processes like adipogenesis and energy balance and coined the term “obesogens” to categorize such chemicals. More specifically, the obesogens hypothesis suggests that exposure to obesogens during development could dysregulate pathways responsible for the development of adipose tissue, alter the metabolic balance and hormonal control of appetite and satiety, and impact insulin sensitivity and lipid metabolism, ultimately resulting in obesity. This hypothesis ignited the field and in 2011, the National Institute of Environmental Health Sciences (NIEHS) initiated the first funding dedicated to obesogens [13]. In the ensuing years, several workshops, reviews, and new research have led to the growth and prominence of this field, with trends continuing to this day.

## 3. Sources of Obesogens

In recent times, humans have been exposed to a vast amount of obesogens present in their everyday lives. According to the Endocrine Society, around 1000 synthetic chemicals are known to be EDCs and interfere with physiological hormonal processes [14]. The most common and well-studied EDCs with obesogenic properties are summarized in Table 1. Exposure can be in the form of plastics and plasticisers, pesticides, personal care products, construction materials, food additives, and packaging, as well as from industrial pollutants found in the air, water, and soil [15]. While many of these sources were initially developed to improve our quality of life and provide modern conveniences, they have become ubiquitous in our daily lives, making exposure to obesogens almost impossible to avoid.

Exposure among children is of greater concern as they are in a critical window of susceptibility and adverse effects can be long-lasting. They also have much greater exposure to obesogens compared to adults as they breathe, eat, and drink more per body surface area, spend more time inside the house where ECDs are more likely to be found, and engage more frequently in object-to-mouth activities [16].

**Table 1:** List of the most common obesogens. This table describes a few of the most important obesogens, their source, and routes of exposure.

EDCs	Source	Route of Exposure
Bisphenol A (BPA)	Used in the manufacture of various plastics such as food packaging, water bottles, water supply pipes, children’s toys, and electronic appliances [17].	Inhalation, ingestion, or dermal contact [17].
Phthalates	Belong to the group of plasticisers used to make plastics more flexible and durable. Found in plastic packaging, children’s toys, personal care products, vinyl flooring materials, clothing, and medical devices [18].	Inhalation, ingestion, or dermal contact [18].
Dioxins	Unwanted by-products of industrial (production of herbicides, smelting or bleaching of paper) or natural processes (forest fires and volcanic eruptions) [19].	The highest level of exposure is through food, especially dairy and meat products, shellfish, and fish. They are mainly stored in the fat tissue of animals, thus accumulating in the food chain [19].
Tributyltin (TBT)	Biocide used to control a broad spectrum of organisms and in marine paints, wood preservation, and industrial water systems [12].	Most common contaminant of marine and freshwater ecosystems. Humans are exposed through inhalation or consumption of contaminated seafood or water [20].
Atrazine (ATZ)	One of the most widely used herbicides in the world; used to control grasses and broadleaf weeds in corn, sugarcane, and sorghum crops [21].	Eating or drinking contaminated products, through inhalation, or dermal contact [21].
Perchlorate	Manufactured for use in fireworks, explosives, rocket fuel, and road flares or naturally occurring in the environment in small amounts [22].	Inhalation, ingestion, or dermal contact [22].
Per- and polyfluoroalkyl substances (PFAS)	Man-made chemicals resistant to heat, water, oil, and grease. Therefore, they are widely used in fire-fighting foams, non-stick cooking pans, food packaging, textile coatings, and household products [23].	PFAS are known to persist in the environment longer than any other man-made chemicals as they break down very slowly. Humans are exposed by consuming PFAS-contaminated food or water, through inhalation of contaminated air, or by direct exposure to PFAS products [23].
Polybrominated diphenyl ethers (PBDEs) and Polybrominated biphenyls (PBBs)	Chemicals used as flame retardants in wire insulation, electronic devices, upholstery, draperies, rugs, and furniture [24].	Ingestion (particularly food high in fat), inhalation, or dermal contact [24].
Triclosan	Used as an antibacterial in products such as soaps, toothpaste, body washes, and cosmetics [25].	Ingestion and dermal absorption [25].
Parabens	Chemicals used as preservatives in food, cosmetics, and pharmaceuticals [26].	Ingestion and dermal absorption [26].
Polycyclic aromatic hydrocarbons (PAH)	Occur naturally in most fossil fuels (coal, gasoline, and crude oil) and are also by-products of incomplete combustion processes [27].	Inhalation of contaminated air (motor vehicle exhaust, cigarette smoke, etc), ingestion (grilled or charred meats or contaminated food), and in some cases dermal absorption [27].

## 4. Pathophysiology

### 4.1. Mechanism of Action

Obesogens contribute to the development of obesity through various mechanisms. Although there is high heterogeneity amongst mechanisms of action, they all share key characteristics. They are recognized for their ability to mimic the function of natural hormones and interfere with the hormonal balance by binding to cellular or nuclear receptors, altering signalling pathways, cellular responses, and gene expression [28]. Their ability to do so depends on the fact that they possess similar chemical features to natural hormones, including low molecular weight, lipophilicity, and long half-lives. These three properties enable them to diffuse into cells more easily, accumulating in the body and competing with the endocrine system [15].

One of the most well-studied mechanisms through which obesogens exert their effects is that of peroxisome proliferator-activated receptor γ (PPARγ) activation. PPARγ is a type of ligand-activated nuclear receptor that plays a crucial role in regulating cellular functions, particularly those related to metabolism and inflammation. It is predominantly found in adipose tissue and regulates the expression of various genes that control adipocyte proliferation and differentiation, glucose metabolism, energy storage, and insulin sensitization [29]. Activation of PPARγ occurs when endogenous ligands, EDCs, or pharmaceutical drugs bind to it, causing it to form a heterodimer with the nuclear receptor 9-cis retinoic acid receptor (RXR). The PPARγ: RXR heterodimer then binds to specific regions on the DNA of target genes called the peroxisome proliferator hormone response elements (PPREs), which cause recruitment of co-transcription factors and subsequent mRNA expression of the target gene [28]. Given its involvement in these metabolic pathways, PPARγ has been an important target for drug development, especially for managing type 2 diabetes and other metabolic conditions. Thiazolidinedione is an example of such a drug whose mechanism of PPARγ activation became evident in the mid-1990s [30]. It is an anti-diabetic drug that works by increasing insulin sensitivity with the expense of inducing adipogenesis and weight gain, thus supporting the proposed mechanism of obesogens contributing to obesity [30].

Furthermore, obesogens do not contribute to obesity through the proliferation of adipose tissue only but also through the formation of ‘unhealthy’ adipocytes, hormone interference, and increasing inflammatory responses. Healthy white adipocytes are characterized by their preserved insulin sensitivity, which facilitates glucose uptake from the circulation and their ability to maintain a balanced inflammatory response, preventing excessive inflammation that can lead to insulin resistance and subsequent metabolic dysfunction. At the same time, healthy adipocytes secrete adipokines such as leptin and adiponectin in appropriate amounts that play a significant role in maintaining metabolic homeostasis. Leptin was first discovered in 1994 by Y Zhang et al. and is a hormone responsible for appetite regulation, glucose uptake in the periphery, and thermogenesis in brown adipose tissue [31]. Similarly, adiponectin was first discovered in 1995 by Scherer PE et al.; two of its key functions are enhancing insulin sensitivity and having anti-inflammatory properties [32]. It has been found that adipocytes produced under the influence of obesogens have an effect on these hormones, thus affecting the hormonal control of satiety and hunger. For example, TBT was found to increase leptin levels and decrease adiponectin levels [33], while di-2-ethylhexyl phthalate (DEHP), the most common type of phthalate, decreased both adiponectin and leptin levels in mice [34]. Similarly, BPA exposure to 3T3-L1 adipocytes in early life was found to increase the levels of leptin mRNA and to disrupt the hypothalamic circuit involved in feeding behaviour and energy homeostasis [35]. All of the above compound the negative effects of obesogens in dysregulating metabolic processes.

Cohesively, chronic inflammation is a common finding in obesity and obesogens can play a role. Apart from the disruption of adipokine balance mentioned earlier, obesogens like DEHP and BPA have been found to promote inflammation in adipose tissue by increasing the production of reactive oxygen species (ROS) and impairing antioxidant defence mechanisms [36]. It has been suggested that when mesenchymal stem cells are exposed to large numbers of ROS, they have a preference to differentiate into adipocytes, thus leading to adipose tissue proliferation [37]. In addition, the imbalance in the production of ROS and their detoxification results in oxidative stress, which can activate inflammatory signalling pathways and damage cellular structures [36]. Moreover, obesogens can induce inflammation by activating and increasing the number of immune cells in adipose tissues such as lymphocytes and macrophages. These, in turn, secrete pro-inflammatory cytokines, like interleukin-6 (IL-6) and tumour necrosis factor-alpha (TNF-a), which initiate and sustain an inflammatory response [15,38].

**Figure 1:**
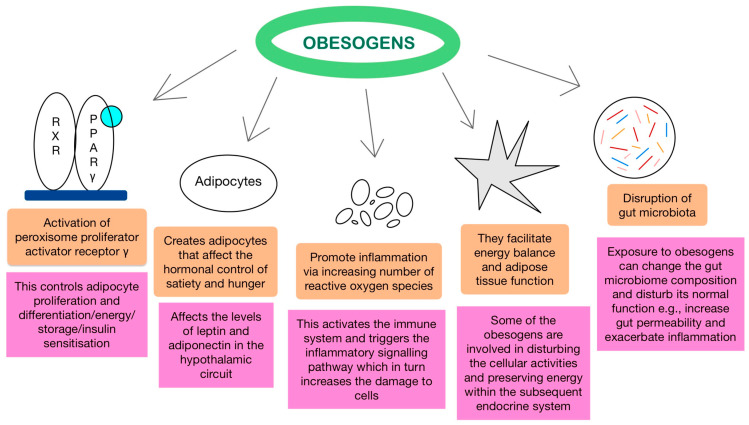
Mechanisms of action of obesogens. The diagram depicts established mechanisms and impacts of exposure to obesogens. This includes the activation of the PPARγ nuclear receptor, hormonal interference, increase in inflammation, and dysregulation of the endocrine system.

Aside from obesogenic interference with leptin and adiponectin, other hormones are also involved. These include androgens and oestrogens, which beyond their reproductive functions, play a crucial role in energy homeostasis and adipose tissue function. The exact mechanisms of action of sex steroids, especially oestrogens, on metabolism and energy balance, are complicated and are out of the scope of this review. In summary, certain obesogens have the ability to bind to oestrogen and androgen receptors to disrupt their cellular activities and the subsequent endocrine system. For example, studies have shown that perinatal exposure of mice to either BPA or DDE leads to heavier offspring and rapid weight gain in infants, respectively, suggesting their xenoestrogenic properties [39,40]. In addition, other studies found that thyroid receptors may also be unintended targets of obesogens. PBDEs, BPA, and phthalates are some of the chemicals with antithyroid properties that can potentially bind to thyroid receptors and interfere with their function. Being a key regulator of metabolism, any alteration in thyroid levels could dysregulate the basal metabolic rate and lead to BMI changes [36,40,41]. The proposed mechanisms are illustrated in Figure 1.

### 4.2. The Role of EDCs on the Gut Microbiota Changes Contributing to Obesity

In addition, it was recently proposed that exposure to EDCs may predispose individuals to obesity by disturbing the gut microbiota. Firmicutes, Bacteroides, Proteus, Actinomycetes, and Fusobacteria make up the majority of the typical human gut microbiome, with Bacteroides and Firmicutes predominating [42]. The main functions of gut microbiota are immunomodulation, antimicrobial protection, nutrient metabolism, and regulation of the intestinal barrier permeability [43]. It is a distinct ‘organ’ containing microbial symbionts that aid in the metabolism of dietary polysaccharides and encourage fat storage, making it a crucial component of energy yield [44,45]. Any modifications in the composition of gut microbiota can interfere with the aforementioned functions and, more specifically, with energy absorption and metabolism. The relationship between the gut microbiome and obesity is well-established. The microbiomes of obese individuals typically exhibit a higher proportion of Firmicutes compared to Bacteroidetes [46]. Firmicutes enhance the body’s ability to extract and store more energy from food compared with other microbes, leading to increased caloric intake and accumulation [47]. Furthermore, transferring the gut microbiome from obese individuals to germ-free mice has been shown to induce obesity in the mice, while microbiomes from lean individuals can promote a lean phenotype and improve metabolic health [48]. Aside from the established link, exposure to obesogens can also change the gut microbiome composition and disturb its normal function. Studies have shown that obesogens, including artificial sweeteners, food additives, dietary emulsifiers, BPA, and TBT can induce gut dysbiosis [49,50,51,52]. This state of imbalance in the gut microbiota has been associated with an increased permeability of the gut barrier, enhancing the likelihood that bacterial endotoxins can cross into the bloodstream and exacerbate inflammation in other parts of the body [53]. This inflammatory state can subsequently give rise to insulin resistance and fat storage, thus predisposing to weight gain [44]. Overall, we can appreciate that alterations in the gut microbiome could be a novel mechanism through which exposure to obesogens promotes obesity, but this mechanism remains relatively unexplored. Understanding and modulating the gut microbiota in the future offers promising strategies for targeting obesity’s underlying mechanisms and improving metabolic health.

### 4.3. Developmental Origins of Obesity

**Figure 2:**
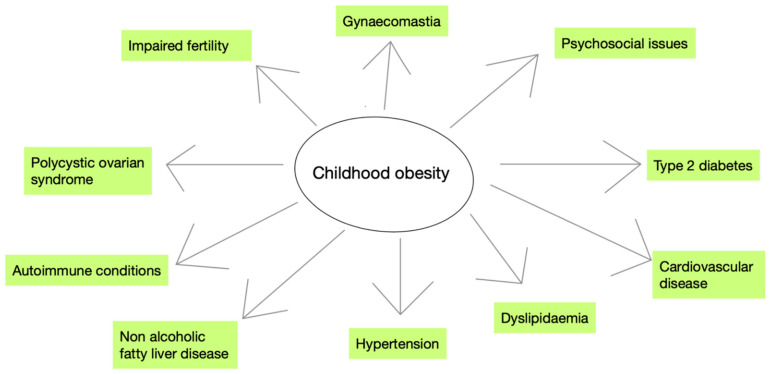
Long-term effects of childhood obesity. This figure summarizes the most important implications of childhood obesity, both physical and psychosocial.

According to the developmental origins of the health and disease hypothesis (DOHaD) there is a connection between foetal environment influences and long-term metabolic effects. Both prenatal and early postnatal life are a pivotal period for child development. Any environmental, maternal, or foetal stressors during this critical window can have profound effects later in life [54]. David Barker proposed a hypothesis that adverse nutrition in early life can increase the risk of metabolic syndrome in adult life. Specifically, he argued that undernutrition during intrauterine life predisposes individuals to conserve calories later in life and increases the likelihood of obesity when exposed to a calorie-rich postnatal environment [55]. A number of studies proved the hypothesis of the role of foetal programming in later life. However, the dramatic rise in paediatric obesity cannot be explained by Baker’s hypothesis alone. Exposure to environmental pollutants during these sensitive periods also comes into play. Numerous animal and epidemiological studies provide evidence supporting the obesogenic impact of early-life exposure to EDCs. One of these is a prospective cohort study involving 412 mother–child pairs from Sweden and Norway, whereby a positive correlation between maternal serum concentrations of perfluoroalkyl substances (PFAS) and higher child BMI was evident at a 5-year follow-up [56]. Similar obesogenic effects have been observed with prenatal exposure to TBT in mice. Offspring of mice exposed to TBT had higher adiposity compared to those not exposed, with similar effects observed in zebrafish, rats, and goldfish [57]. Additionally, another study revealed that higher levels of phthalates detected in maternal urine during pregnancy were associated with a twofold increase in the probability of the offspring being obese [36]. Likewise, maternal exposure to BPA and DDT during pregnancy has been correlated with higher BMI, waist circumference, and risk of obesity in offspring [58,59]. Long-term consequences include an increased risk of developing chronic health conditions such as type 2 diabetes, cardiovascular disease, dyslipidaemia, and hypertension. Specifically, research shows that at the time of diagnosis, more than 85% of children with type 2 diabetes are overweight or obese [60], and 30–50% of obese adults were also obese during childhood, suggesting that the cardiovascular risk factors usually persist in adulthood [61]. Furthermore, 28–41% of obese children and adolescents are found to have non-alcoholic fatty liver disease (NAFLD), making it the most common liver condition in childhood [62]. Additionally, childhood obesity has been associated with increased prevalence and worse prognosis of certain autoimmune conditions such as type 1 diabetes, inflammatory bowel disease, rheumatoid arthritis, psoriasis, and systemic lupus erythematosus [63]. Moreover, obesity increases the risk of polycystic ovarian syndrome (PCOS) in females, gynecomastia in males, and reduced fertility in both [63]. All of the above highlight the dangerous effect of environmental chemicals as obesogens and their impact on human health, particularly during critical periods of development. Despite the numerous adverse effects on physical health, the psychosocial issues that can arise with childhood obesity should not be underestimated. Children and adolescents who are overweight or obese often encounter challenges with self-esteem and may experience feelings of depression [63]. They are vulnerable to bullying at school, with a study confirming that around 45–50% of obese adolescents experience frequent bullying [64]. Additionally, academic performance may be adversely affected, with overweight or obese children typically achieving lower grades and attaining a lower level of education compared to their peers of normal weight [63,65]. The aforementioned long-term effects of childhood obesity are summarized in Figure 2.

## 5. Epigenetics

The term epigenetics was first introduced in the 1940s by Conrad Waddington and is currently referred to as the study of changes in gene expression that occur without alterations in the underlying DNA sequence i.e., not the result of mutations [66]. These changes can be influenced by various factors, including exposure to EDCs or obesogens during pregnancy or during the early postnatal period. Multiple mechanisms of epigenetic change have been proposed, including DNA methylation, histone modification, and altered microRNA expression [67]. DNA methylation is the most commonly studied mechanism and involves the addition of a methyl group to a specific region of DNA, thereby impacting gene expression. Exposure to obesogens during critical windows of development can lead to changes in the expression of genes associated with adipogenesis and metabolism [68]. For example, a recent study found that methylation of the cg11531579 island site (CHFR) is associated with accelerated weight gain in early life and the development of obesity [69]. In addition, exposure to obesogens can disrupt histone modifications, which regulate the structure of chromatin and gene expression. If genes involved in metabolic regulation are targeted, the risk of obesity later in life increases [68]. TBT is an example of an obesogen found to induce changes to histone methylation in vitro [68]. Similarly, exposure to obesogens can change the expression of miRNAs that are important for lipid metabolism, insulin signalling, and adipocyte differentiation, all of which can lead to the development of obesity [67].

The changes in phenotype that occur secondary to epigenetic modifications can manifest soon after exposure to EDCs or later on. The timing of exposure is particularly important in determining the extent of these changes [70]. On the one hand, if exposure and epigenetic changes occur during early development, an irreversible change occurs in the germline’s epigenome, potentially passing on the modified epigenome to future generations. To truly exhibit transgenerational effects, these phenotypes should be evident in the F3 generation, as this would be the first generation that was not directly involved in EDC exposure [71,72]. On the other hand, if epigenetic changes secondary to EDC exposure arise in adulthood, they usually affect the individual’s somatic cells and are neither hereditary nor permanent. Moreover, it is crucial that both the specific tissue and dose of EDCs are taken into consideration before drawing definite conclusions regarding their epigenetic effects [67]. Overall, epigenetic mechanisms provide a framework for understanding how obesogens influence gene expression and metabolic processes to promote obesity.

### The Impact of the Food Industry on Obesity

Given the rise in global consumption of fatty and sugar-rich foods, it comes as no surprise that recent trends in the prevalence of obesity have been on the rise. The CDC estimates that in the USA and its territories alone, more than 1 in 5 people are reported to be obese [73]. However, as previously mentioned, these rising trends might not be attributed to energy imbalances alone, external factors such as the presence of obesogens in food may play a vital role in understanding the nuances behind childhood and adolescent obesity. Phthalates, which are known obesogens, have been used since the 1930s as additives in plastics, improving their elasticity, workability, and extensibility, thereby making these plastic polymers an excellent candidate for food packaging. That said, mounting concerns have been rising as phthalate contaminants have been increasingly making their way into the packaged foods [74]. Cohesively, numerous studies have revealed statistically significant correlations between exposure to phthalates and the development of insulin resistance, metabolic syndromes, and obesity [74,75]. As of 2020, in an effort to be environmentally friendlier, companies in the fast-food industry have implemented new forms of packaging that use less plastic; recent studies concluded that the level of phthalates present in said packaging was of low enough concentrations that it did not significantly contribute to the overall consumer exposure to phthalates [76,77]. However, other current studies show that the consumption of fast food is still associated with increased exposure to phthalates [77]. The contradictory nature of these results could potentially be attributed to a multifactorial cause, from the bioaccumulation of obesogens introduced during food processing and from the environment [74], to the temporal accumulation of obesogens over time through consistent consumption of ultra-processed foods [78]. Building off that, it is imperative that exposure to obesogens such as phthalates not be attributed to a single isolated source such as fast-food consumption alone. In fact, other sources of obesogens can come from the aforementioned ultra-processed foods, which are defined as products comprised of multiple ingredients and additives that are made through biochemical and physical processes where whole foods become mostly or completely absent [78], are nutritionally unbalanced, or energy-dense [79].

Ultra-processed and fast foods mainly appeal to children and adolescents due to their convenience, ease of availability, and aggressive marketing strategies adopted by companies selling them [80,81]. Therefore, it can be assumed that due to the target audience of these products, a statistical rise concurrent with the CDC’s estimations would be observed on a global scale. Multiple studies from the current literature maintain that diets rich in ultra-processed and fast foods have been significantly associated with a higher prevalence of obesity, and these trends alarmingly persist on a universal scale; Saudi Arabia [82], Australia, China [83], South Asia [84], South-East Asia [85], and North America [86] have all demonstrated the impact of the fast food industry on public health with alarmingly congruent results, indicative of a correlational effect between the consumption of fast and ultra-processed foods and a rise in the incidence rates of obesity and cardiometabolic risk factors [79]. Quintessentially, these findings underscore the need for heavier regulations on the way ultra-processed and fast foods are marketed to the younger generations. Some studies suggest regulating these food products similarly to alcohol and tobacco, albeit to a lighter extent, such as implementing tax legislation to deter their overconsumption. Though some countries tax certain products such as sodas at around 10%, researchers at an Oxford group predict that this number should be at least as high as 20% to have a significant effect [87].

## 6. Prevention Strategies

Preventing obesity nowadays is a great challenge, as Hippocrates said, “preventing is better than cure”. The World Health Organization has caused alarm by recently releasing a new obesity prevention framework that endorses the WHO Acceleration Plan to Stop Obesity [88]. The key aim of this strategy is to recognize obesity as a chronic disease and to replace models of obesity comorbidity management with models of obesity prevention starting from early childhood. The framework supports the integration of primary health carers to play a pivotal role in obesity prevention as the frontline health care providers. This is important as prevention must start in infancy or early childhood, but also much earlier with prenatal programming.

Preventing and managing obesity is no easy task due to previous neglect of the disease individually or/and globally for many years. Multi-sectoral and multi-thematic actions are needed in order to achieve the task; governmental, societal, and individual measures must be adopted. The recognized environmental impact of obesogens on the development and progression of obesity has led to the incorporation of specific actions and policies regarding the control of the EDCs that act as obesogens. Nevertheless, years ago, Hippocrates recognized that the quality of food has a reflective impact on human health by saying ‘let food be thy medicine and medicine be thy food’.

### 6.1. Individual Actions

Individuals and families must follow a balanced diet and include physical activity in their everyday lives, and canned and packaged food consumption must be reduced to a minimum. The use of cosmetics containing triclosan, dioxins, parabens, and other EDCs should also be avoided [89]. Plastic materials in general and plastic food containers, in particular, must be avoided especially prenatally and during neonatal life, as plastics can affect not only the child’s metabolism but also neurodevelopment [90]. Specifically, families must focus on purchasing labelled phthalate-free products and avoid microwaving or heating food in plastic containers. The US Food and Drug Administration (FDA) also advices that all damaged plastic bottles be discarded, as they may harbour bacteria and lead to the release of more BPA. In February 2024, the FDA announced the removal of EDCs per- and polyfluoroalkyl substances (PFAS) from food packaging [91]. Individuals must read food container codes in order to avoid such substances. Finally, all individuals must consume organic fruit, vegetables, and grains.

### 6.2. Healthcare Providers’ Actions

Healthcare providers have a leading role as the frontline carers of children and families. They need to teach families about the importance of a healthy lifestyle throughout life, starting as early as the prenatal period [92]. Additionally, obstetricians must advice future mothers to avoid contact with EDC lotions, cosmetics, and hair care products, and also advise them to eliminate contact with foods in plastics, as well as processed foods. Paediatricians must underline the importance of lifestyle modifications, including balanced food consumption and physical exercise, as early as possible to all children. Paediatricians should especially emphasize the aforementioned to families at high risk of obesity and its complications due to genetic predispositions [93]. Cohesively, researchers can help by developing models for early recognition of chemical exposure using genomic studies in order to enhance prevention strategies [94].

### 6.3. Community Actions

Societies and governments must first recognize the relationship between the obesity pandemic and its associated risks with the early exposure of EDCs in childhood, as environmental obesogens such as thiazolidinediones, organotins, perfluorooctanoic acid, diisobutyl phthalate, and bisphenol A are still being used worldwide. Urgent development of policies to ban these substances and remove them from the environment must be adopted. Actions at a regulatory level are also important for industries that use EDCs in their products to be banned from the market. Labelling food products, cosmetics, and plastics with ingredients containing EDCs shall inform and warn consumers. European countries have started a practice of becoming ‘greener’ by banning BPA in food packing materials in schools and eliminating the use of obesogenic chemical substances in their industries [95].

Approximately 1000 synthetic compounds were identified as EDCs in contemporary society, with 50 being obesogens [54]. After analysing some of the most important obesogens and looking into their source and exposure route, we can confidently say that exposure is widespread. They are found in various everyday items such as plastics, pesticides, personal care products, and food additives, which makes them hard to avoid given the ubiquity of these sources. To reiterate, children are particularly vulnerable as they are in a critical developmental stage; the issue is further compounded given their higher levels of exposure due to behaviours such as mouthing objects and the fact that they spend more time indoors where EDCs are more prevalent.

## 7. Conclusions

This literature review delves into the complex issue of childhood obesity, highlighting its significant rise globally and the multitude of factors contributing to its prevalence. The identification of endocrine-disrupting chemicals acting as obesogens and their potential contribution to the obesity epidemic, beyond the prevailing medical explanation of energy imbalances, represents a significant paradigm shift in understanding the aetiology of this multifactorial condition.

In conclusion, this literature review underscores the urgent need for a holistic approach to combat childhood obesity, acknowledging the multifactorial nature of the condition and the critical role of environmental factors such as obesogens. By addressing the complex interplay between genetic predisposition, environmental exposures, and lifestyle factors, stakeholders can work towards implementing effective prevention strategies and mitigating the long-term health consequences associated with obesity.

## 8. Future Directions

While there have been significant advancements in understanding the different types, number, and mechanisms of action of obesogens, there still remains a considerable amount to discover regarding their overall impact on obesity vulnerability. One area in which the current literature is lacking is how obesogens may interact with nutrients in the diet to promote obesity. In addition, the influence of obesogens on the gut’s microbiome composition and its contribution to obesity is not well understood and may potentially offer innovative approaches for treating obesity. Another challenge is comprehending the effects of exposure to a combination of obesogens—whether they produce additive or synergistic effects or hinder each other’s actions. In essence, all these important questions need to be addressed as this knowledge will be instrumental in devising strategies to prevent or minimize exposure to these harmful substances. The European Union made an important effort by funding eight international consortia through its Horizon 2020 grant program to examine EDCs, three of which specifically focus on developing techniques to identify obesogens [68,96]. These efforts are expected to yield fruitful results as they aim to identify the complete range of obesogens and understand their mechanisms of action. Moreover, although the use of animal models and screening assays have so far been fundamental, results cannot be extrapolated confidently to humans. Thus, in the future, it will be beneficial to aim for longitudinal studies on humans, which will provide a more robust dataset with more valid results. Ultimately, all of the aforementioned initiatives seek to provide healthcare professionals and the public with a thorough understanding of the dangers associated with obesogen exposure. This will enable them to make informed decisions and implement changes that will minimize exposure and mitigate health risks, combating the obesity pandemic.
